# Barriers to Timely and Safe Blood Transfusion for PPH Patients: Evidence from a Qualitative Study in Dhaka, Bangladesh

**DOI:** 10.1371/journal.pone.0167399

**Published:** 2016-12-02

**Authors:** Sadika Akhter, Iqbal Anwar, Rashida Akter, Feroza Akhter Kumkum, Monjura Khatun Nisha, Fatema Ashraf, Ferdousi Islam, Nazneen Begum, Mahbub Elahi Chowdhury, Anne Austin, Syed Shariful Islam, Aminur Rahman

**Affiliations:** 1 International Centre for Diarrhoeal and Disease Research Bangladesh (icddr,b), Dhaka, Bangladesh; 2 Centre for Environment and Population Health, Griffith University, Australia; 3 School of Public Health, Sydney Medical School, Sydney, Australia; 4 Shaheed Suhrawardi Medical College and Hospital, Dhaka, Bangladesh; 5 Dhaka Medical College and Hospital (DMCH), Dhaka, Bangladesh; 6 JSI Research & Training Institute, Inc. Boston, MA; 7 Department of Public Health and Informatics, Bangabandhu Sheikh Mujib Medical University, Dhaka, Bangladesh; 8 College of Public Health Sciences, Chulalongkorn University, Bangkok, Thailand; National Institutes of Health, UNITED STATES

## Abstract

**Background and Objectives:**

In Bangladesh, postpartum hemorrhage (PPH) is the leading cause of maternal mortality accounting for 31% of all blood transfusions in the country. Although safe blood transfusion is one of the 8 signal functions of Comprehensive Emergency Obstetric Care (CEmOC) strategy, most of the designated public sector CEmOC facilities do not have on-site blood storage system. Emergent blood is mainly available from external blood banks. As a result, emergent patients are to rely on an unregulated network of brokers for blood which may raise question about blood safety. This study explored lived experiences of patients’ attendants, managers, providers, and blood brokers before and after the implementation of an on-line Blood Information and Management Application (BIMA) in regards to barriers and facilitators of blood transfusion for emergent patients.

**Methods:**

Data were collected at Dhaka Medical College Hospital (DMCH), a tertiary-level teaching hospital before (January 2014) and after (March 2015) the introduction of an online BIMA system. Data collection methods included 24 key informant interviews (KIIs) and 40 in-depth interviews (IDIs). KIIs were conducted with formal health service providers, health managers and unlicensed blood brokers. IDIs were conducted with the relatives and husbands of women who suffered PPH, and needed emergency blood.

**Results:**

Patients’ attendants were unaware of patients’ blood type and availability of blood in emergency situation. Newly introduced online BIMA system could facilitate blood transfusion process for poor patients at lower cost and during any time of day and night. However, service providers and service recipients were heavily dependent on a network of unlicensed blood brokers for required blood for emergent PPH patients. Blood collected through unlicensed blood brokers is un-screened, unregulated and probably unsafe. Blood brokers feel that they are providing a needed service, acknowledged a financial incentive and unaware about safety of blood that they supply.

**Conclusions:**

Ensuring safe and timely blood transfusion is necessary to end preventable maternal mortality. In a context where facilities have no on-site blood, and both providers and patient attendants are heavily dependent on an unregulated cadre of unlicensed blood brokers, access to timely safe blood transfusion is seriously threatened. BIMA is a promising intervention to reduce inefficiencies in obtaining blood, but steps must be taken to ensure buy-in from current purveyors of blood, and to increase the acceptance of the intervention.

## Introduction

Although Bangladesh has made remarkable progress in achieving Millennium Development Goal 5 (MDG5) targets, [1] many women still die of preventable maternal causes[2]. The country is the tenth largest contributor to the global maternal death burden, with an estimated 5,500 maternal deaths in 2015[3]. Sustainable Development Goals (SDG) call for a global reduction in MMR to 70 or less maternal deaths per 100,000 live births by 2030 (SDG goal 3.1)[4]. However, the current MMR in Bangladesh is 176 death per 100,000 live-births where postpartum hemorrhaged (PPH) is the single leading cause of maternal deaths.[5]. Saving the life of PPH patients require early recognition and emergency response with a range of technical procedures including organizing safe blood transfusion in a timely fashion.[6].

Ensuring timely access to safe blood for women suffering from PPH is challenging in Bangladesh. Although safe blood transfusion is a key component of any comprehensive emergency obstetric care (CEmOC) strategy, majority of designated CEmOC facilities in the public sector do not have any on-site blood storage facilities[7]. Patients are to rely on external sources for emergency blood. In addition to government blood banks (attached to secondary and tertiary level government hospitals), a number of NGOs have blood banks to support safe blood transfusion such as Bangladesh Red Crescent Society, The Quantum Foundation, Badhon and the Sandhani. Sandhani is a philanthropic organization run by medical students to support blood transfusion process for poor admitted patients in government hospitals and works in close collaboration with government blood banks situated within hospital premises. While other NGOs organize blood independently, and are situated outside government hospital premises. The government has formulated guidelines on blood safety, however they are poorly implemented on the ground due to acute shortage of trained human resources, reagents and apparatus, and insufficient supervision and monitoring[8, 9].

We conducted a mixed method intervention study to facilitate safe blood transfusion process for admitted PPH patients in Dhaka Medical College Hospital (DMCH), a tertiary level public-sector teaching hospital in Dhaka, Bangladesh. This intervention included development and introduction of an online blood information management application (BIMA) system to reduce delay in getting blood by emergent patients in obstetric wards. The developed online BIMA system allowed easy online access to two different blood information databases within the hospital. The BIMA system captures the information regarding the name and location of the blood centres, the current stock of blood units, donor list (with group & Rh typing), the availability of particular groups of blood and booking information for required blood [10]. BIMA connected the obstetric wards of DMCH with two proximate blood banks (government and *Sandhani*) situated within the hospital premises.

The mixed method intervention study followed a before-after research design where the quantitative study tested the effectiveness of BIMA intervention in reducing delay in accessing blood for PPH patients and the qualitative component explored insights into the ‘lived’ experience and perceptions of people involved in emergent blood acquisition process. This paper presents findings from the qualitative study highlighting barriers and facilitators for obtaining emergent blood from the perspectives of health care providers, patient attendants, and unlicensed blood brokers.

## Methods

We collected qualitative data through in-depth interviews (IDIs) and key informant interviews (KIIs) and in two phases; during baseline (January to March 2014) and end line (November-December 2014) assessments. A team of researchers with backgrounds in anthropology and training in qualitative research methods conducted interviews. Consent forms and separate open ended interview guidelines were prepared for conducting KIIs and IDIs [S1 File]. We tested the developed interview guidelines in a hypothetical field (in a different medical college hospital in Dhaka city). Based on field-testing, the guidelines were revised for final data collection. Voice recorders were used to minimize data lost.

**Fig 1:**
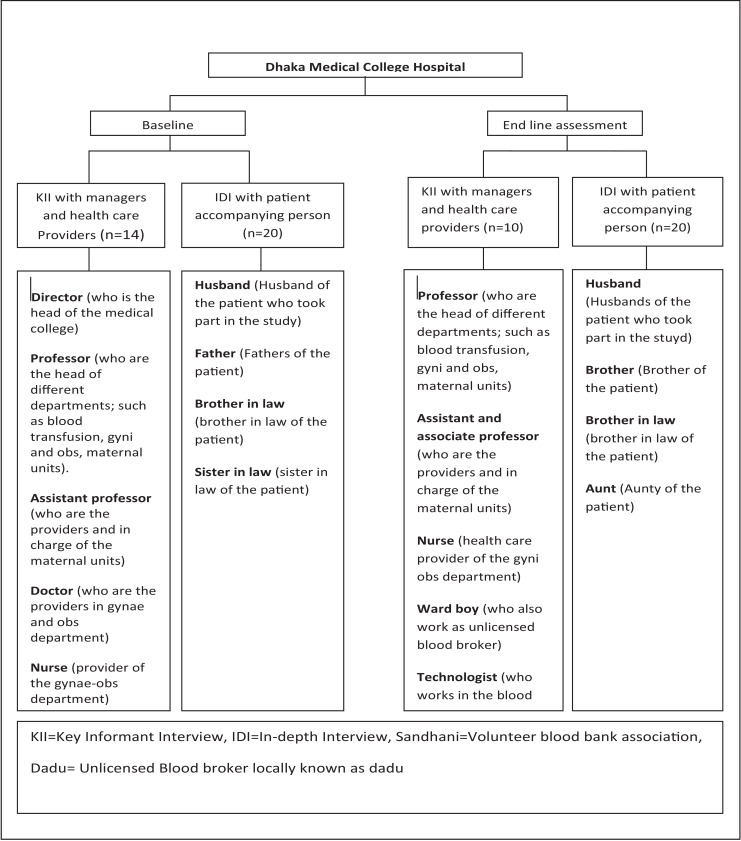
Detail sampling frame

We conducted 24 KIIs and 40 IDIs in total during pre and post intervention period (Fig 1 and Table 1). KIIs were conducted with hospital directors, managers, providers, blood bank authorities and unlicensed blood brokers to understand how emergent blood were acquired before and after the introduction of BIMA. IDIs were conducted with patients’ relatives to understand their perception and experiences of emergent blood collection process before and after the BIMA intervention. We used purposive and snowball sampling methods to identify study participants.

**Table 1:** Summary of sample size

Study participants	Sample size Baseline assessment	Sample size end line assessment
KII with hospital director, professor cum managers	07	01
KII with associate professor and assistant professor	02	03
KII with healthcare providers, nurses and blood broker	04	03
KII with blood bank executive, technologists	01	04
IDI with patient relatives	20	20
Total	34	30
Grand total	64	

The interviews took place mainly in Dhaka Medical College Hospital or at a suitable place of respondent’s choice; interviewers tried to secure a setting with minimum disturbances. Each interview lasted between 40–60 minutes. We used an ID number for each respondent to ensure anonymity. After finishing interviews, the researchers prepared verbatim transcriptions of each recorded interview. All the interviews were conducted in Bangla. The supervisors and study investigators closely monitored the interview transcription process to check fidelity. Data were coded and analyzed in Bangla.

### Data analysis

Data were coded to categorize the key concepts for analysis [11]. Codes were developed after reading and re-reading the textual data generated through KII’s and IDI’s. Authors, who are trained in qualitative research and data analysis, agreed on a set of codes to apply to all transcripts. Themes were developed based on the frequent and uniform statements of the individual study participants. [12, 13]. Codes were categorically grouped to conduct a thematic analysis. Data were systematically indexed, summarized and interpreted to understand the explanations of the study findings. Results from respondents were compared to strengthen the validity of the findings.

### Ethical Approval

Verbal informed consent as well as written consent was obtained prior to each interview from all study participants. Every respondent was given adequate information on why he/she was invited to participate, his/her role, and potential risks and benefits of participating in the study. Study participants were also informed of steps taken to ensure their confidentiality, privacy and anonymity. Participants were informed of their right to withdraw from the interview session at any time they wanted.

Ethical approval (protocol number -13048) was obtained from both the Institutional Review Board (IRB) of icddr,b and Dhaka Medical College Hospital (DMCH) prior to implementation of the study.

### Results

#### Background characteristics of the study participants

Of total 24 key informants, 20 were aged 30 years and above, 16 were females, and 23 were Muslims (Table 2). Fifteen of them had a MBBS degree, eight had completed grade 10 and above education and only one respondent had no formal education. By profession, they were directors, professors, assistant professors, medical doctors, assistant registers, nurses, and unlicensed blood brokers, medical technologists and formal blood bank employees.

**Table 2:** Socio demographic information of key informants

Socio–demographic status of the KII	Base line assessment	End line assessment	Total
Age			
Below 30 years	02	02	04
30 or above	12	08	20
Total	14	10	24
Sex			
Male	03	05	08
Female	11	05	16
Total	14	10	24
Religion			
Muslim	14	09	23
Hindu	00	01	01
Total	14	10	24
Education			
No formal education	01	00	01
Class 1 to 4	00	00	00
Class 10 and above	03	05	08
MBBS and above	10	05	15
Total	14	10	24
Designation			
Director	01	00	01
Professor	02	00	02
Associate professor	03	01	04
Assistant professor	00	02	02
Doctors	04	00	04
Assistant register	00	01	01
Nurse	02	01	03
Unlicensed blood broker	01	01	02
Medical technologist	01	00	01
Private Blood bank employee	00	01	01
Total	14	10	24

Of total 40 IDI participants, majority (n = 31) were aged 30 years and above, thirty five were male and 39 were Muslim (Table 3). Of them 18 completed grade 5–9 education; 7 completed 1–4 grades, 6 participants completed grade 10, and 9 participants had no formal education. IDI participants were from different occupational backgrounds such as housewives, rickshaw pullers, house-maids and garment factory workers, and others.

**Table 3:** Socio-demographic information of IDI respondents

Socio-demographic status of IDI	Base line assessment	End line assessment	Total
Age			
Below 30 years	04	05	09
30 or above	16	15	31
Total	20	20	40
Sex			
Male	19	16	35
Female	01	04	05
Total	20	20	40
Religion			
Muslim	19	20	39
Hindu	01	00	01
Total	20	20	40
Education			
No formal education	00	09	09
Class 1 to 4	05	02	07
Class 5 to 9	10	08	18
Class 10 and above	05	01	06
Total	20	20	40
Occupation			
Housewife	01	02	03
Agriculture	01	02	03
Driver	01	04	05
Business	06	04	10
Rickshaw Puller	01	01	02
Unemployed	01	01	02
Retired	01	00	01
Carpenter	01	00	01
Service	05	00	05
Mason	01	00	01
Mechanic	01	00	01
Garment worker	00	02	02
Private job	00	03	03
Housemaid	00	01	01
Total	20	20	40

### Managing of Blood as lived experiences: voices of study participants

#### No knowledge of where to find blood when doctors identify the need

When someone comes to DMCH with a PPH patient, immediately asked to manage blood by the on-duty doctor or nurse. Most of the respondents stated that managing blood was challenging as they did not know where to go for blood. One patient attendant mentioned *“the nurse told me to get blood*. *But I don’t know how to manage blood!”* The doctors or nurses did not give them any instruction about where to go and how to organize blood. The instruction from doctor simply was to obtain blood as soon as possible. As one respondent said, *“After we arrived at the hospital*, *the doctor told us to obtain blood from ‘the new building’*. *We don’t know the new building*, *we came from a village*.*”*

#### No knowledge of the need to bring a blood donor

At DMCH, it has become the norm for doctors to expect patients to bring their own living blood donors. However this is not something patient attendants are aware of prior to admittance. Attendants indicated that either they didn’t have any donor, or they were unaware about the need of a blood donor before coming to the hospital. As respondent said, “We came to know that we need to collect blood urgently for our patient after we arrived at the hospital. If we would know beforehand about the need of blood, we would bring a blood donor.” Another mentioned, “*It was Friday*, *there was nobody to ask for help*. *We didn’t have our own people to give blood for the patient*. *The doctor told us that if blood was not given within an hour*, *the patient will die…*. *we passed two hours without blood as we don’t know where to go for blood*.*”*

The health care providers also indicated that population are unaware about importance of bringing their own donor for blood transfusion. Most of the respondents stated that they come to the hospital without a donor.

The study revealed that though few donors were available with PPH patients, they simply declined to donate blood due to fear of untoward effects blood-donation. In one case, even the father did not want to give blood for the daughter due to the fear. Many attendants stated that they believed that “*if they give blood*, *they will die*.*”* Some doctors opined that these misconceptions create obstacle to collect blood from the relatives of the patient. One doctor noted that, *“We found that the donor of the patient escaped the hospital out of fear that he might be requested to give blood*. *We do not have a blood counselor at the hospital*. *We need a blood counselor to counsel the donor to encourage giving blood*.*”*

### The role of unlicensed blood brokers

#### Managing blood

During baseline study, it was found that the most common method of obtaining emergent blood was through an unlicensed blood broker. When attendants were asked to collect blood for their patients after arriving at the hospital (without a blood donor), the doctor/nurse introduced them to a unlicensed blood broker. These unlicensed blood brokers were able to organize blood quickly, for a fee. One husband noted, *“After the delivery of my wife*, *the doctor told me to collect blood for my wife*. *I asked the doctor how I can get blood*. *The doctor told me to go to the unlicensed blood broker*, *who was in the ward…*. *and he (the doctor) drew blood from my wife to test the blood group*. *He came-up within few minutes and informed me that her blood group is B+*. *The blood broker brought blood within an hour and I paid him 1600 taka for the blood*. *I don’t know from where he got the blood*. *I don’t know whether the blood is good or bad*.*”*

Another attendant explained, *“After the admission of my patient at hospital*, *the doctor told me to collect six bags of blood*. *The doctor also said to manage blood at any cost*. *A man from the ward came to me and said to me that he will manage blood for me*. *He took me to a place by a rickshaw and gave me two bags of blood and I paid him 2700 taka for two bags of blood*. *It took half an hour to get blood through unlicensed blood broker*.*”*

#### Doctors depend on unlicensed blood brokers in emergent situations

The doctors working in the obstetric wards of DMCH also indicated that they needed blood brokers to organize blood in emergency situations. According to the doctors, they first request that patient’s attendants to obtain blood from their own relatives. If the attendants fail to find a blood donor, doctors send a request for blood to the hospital blood bank. When blood bank is unable to provide requisite blood, doctors depend on the unlicensed blood brokers to obtain blood. One doctor mentioned, *“We cannot ignore them (unlicensed blood brokers)*, *rather we depend on them to get blood to save life of a patient*.*”* In this regard another doctor said, *“When we need blood within half an hour to save life of a patient*, *we said the patient to collect blood immediately with the help of unlicensed blood brokers*. *They collect blood from a professional donor to save life*.*”*

#### No way to assess the quality/type of blood

Doctors mentioned that often they do not have time to screen blood obtained though the unlicensed brokers, as many patients come to the hospital in a very critical condition and are in need of emergency blood. As one doctor described, *“Most of the patients come here in very critical condition and they need blood transfusion within an hour*. *We start treatment with giving an intra-venous saline and wait to receive blood*. *We need blood urgently*. *We could only cross match the blood type for safe blood transfusion but we cannot ensure the quality of the blood*. *As a result*, *we had some incidents of the death of the mothers in our gynecology word due to the unsafe blood transfusion*.*”*

A majority of the relatives who collected blood through unlicensed blood brokers stated that they do not have any idea about the quality of the blood collected by the blood broker from outside the hospital. Some of the respondents went with the broker to an unknown place to get blood and some of the respondents received the blood from the broker at the hospital premises. The blood collected from outside were transfused mostly without screening. One respondent said, *“It would be good if we would get the blood from the blood bank of the hospital*. *I got blood outside of the hospital*. *I don’t know whether it was good or bad blood*. *The addicted people sell blood*. *This blood is polluted*.*”*

The respondents also indicated that it is very difficult to get fresh blood as donors are not available. They are to give four-five days old blood to patients, as the blood is collected from the professional donors. The hospital cannot ensure the quality of the blood as it is given without screening. Doctors were doubtful of the safety of blood that is collected by the unlicensed blood brokers, noting that that the blood was unscreened, and therefore not safe.

Unlicensed blood brokers also indicated that they don’t know anything about the safety and quality of blood. One of the unlicensed blood brokers said, “*humm*….*the blood from outside supposed to be bad (it means the quality of blood) and it should be*. *I don’t know from whom the private blood bank collected the blood*.*”*

#### Cost of blood collected from an unlicensed blood broker and financial incentive

The study participants mentioned that the price of blood varies from place to place; if from government blood bank or NGO sources (Sandhani or Quantum Foundation), the cost of blood is 500–800 BDT. While the price of one unit blood ranges from 800 to 1600 taka through unlicensed blood brokers. The price is higher when the blood group is negative. It takes around 2500 taka.

One respondent said, *“It cost minimum 1000/1200 taka to collect one bag blood for the patient*. *It cost 250 taka to cross match the blood*. *If the blood is negative group*, *it cost 2000–3000 taka*. *There are some other costs such as blood screening and blood group test cost*.*"*

The interview with the blood broker indicated that they get commission from the private blood bank but they did not disclose the amount they receive as commission. They also admitted that they often get some additional money from the patients relatives as tips.

#### Unlicensed blood brokers feel that they are providing a very important service

Blood brokers felt that that they were providing an important service to emergent patients when their attendants fail to organize needed blood immediately. Blood brokers work in the hospital wards, and keep an watchful eye when patients’ relatives need blood. Blood brokers offer their services in dire emergencies when patient attendants have no easy alternative. A number of respondents who collected blood through unlicensed blood brokers mentioned that it did not take long time to collect blood through them. The data revealed that in most of the cases the unlicensed blood broker could manage blood within half an hour, except if a patient needed Rh-Negative blood; it takes more than an hour to collect blood of RH-negative blood.

One unlicensed blood broker stated, “*The patients come to the hospital when their relative/attendants are in danger with their patient*. *Then they need help of blood*, *they need a lot of blood and they need it urgently*. *Urgently means the blood bank like Sandhani and Quantum they don’t sell blood*. *They take long time to give blood*. *You need to be in the queue for blood even more than ninety minutes to know about the availability of required blood if you want to collect blood through Sandhani and Quantum (licensed blood bank)*. *The condition of patient is already critical; if it takes long time to get blood the condition of patient will be more critical and she will die*. *There are three-four private blood banks near the hospital*, *so we bring blood to save life of patient very quickly from those blood banks*.*”*

#### Strong relationships with professional blood donors/blood banks

**Fig 2:**
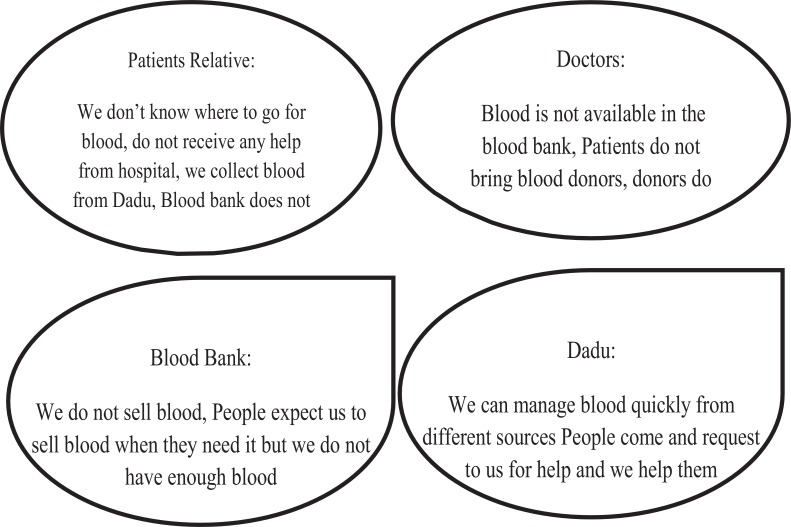
Different voices from different stakeholders

The interviews with the unlicensed blood brokers also revealed their strong networks and linkages [Fig 2]. The data suggests that this unlicensed blood brokers who work at the hospital as “ward boy” have very strong liaison with the nearby unlicensed private blood banks, professional donors, and with the staffs working in licensed blood banks (public or NGO). They explained that their unethical relationship with the staff who work at the hospital blood bank help in creating artificial shortage of blood there and that compelled to buy unsafe blood from outside unlicensed sources with high price.

### Blood Information Management Application (BIMA)

#### Benefits

Doctors working in the hospital blood bank clearly indicated that there was communication gap between the hospital doctors and the blood bank doctors before implementation of BIMA. Staff s who works at the hospital ward didn’t know which group of blood was available in the blood bank (government and Sandhani). When ward doctor or nurse sent requisition for a certain group of blood, if not available in the blood bank, the relatives of the patient do not want to understand the situation. Relatives and attendants used to believe that there was blood in the blood bank but blood bank staffs did not want to provide blood.

The digital system could update the record of the availability of blood each morning and it worked closely with ward and blood bank. Whenever a request was raised anyone could check the availability of blood from the updated list and contact with the blood bank staff directly for emergency need of blood.

After implementing BIMA, interviews with the relatives of the patients and their attendants found that it takes less time now to manage blood due to the digital blood collection system. It further found that it is less hazardous to manage blood during emergency situations. In this regard one respondent mentioned *“It was a great help*, *I would not be able to arrange blood so easily with a short notice*.*”*

Another informant who received blood through the digital blood management system also expressed his satisfaction. He mentioned *“We do not have to wait or run here and there for blood*. *It saved the life of our patient*. *It would be good if everyone can get the information about this program*.*”*

#### Barriers

The BIMA system was introduced in the obstetrics wards to provide information on the availability blood for patients in the hospital blood banks, who needed blood for treatment. help desks were organized with study research assistants round the clock in the obstetrics wards to help PPH patients in getting blood quickly using online BIMA system. They had the updated information on availability of blood as per blood groups stored in Sandhani and in the government blood bank. We asked the users how they got the information regarding the digital system. They replied that there was no sign board or anything about the service. Almost all of them perceived that there could be a big sign board up-front so that they could get the information on availability of blood from the reception/admission desk.

Another challenge was frequent transfer of trained staff. Study participants stated that trained staff used to get transferred to another department. Medical officers, honorary and other staff are changed quite frequently. Those who were trained on the digital blood information services system did not communicate to the newly appointed ones. There is no mechanism to follow up this.

The respondents indicated as challenge that it is difficult to get blood after office hours specially when it is midnight. It was also found that Sandhani is closed during after office hours particularly during nights.

#### Lack of knowledge about BIMA

Patients were inadequately informed about developed digitalized BIMA system. A number of doctors were also not aware about digitalized system. Moreover, obstetric ward doctors had good relation with unlicensed blood brokers, and they were dependent on unlicensed blood brokers for emergent blood. In some cases they were reluctant to use the digital blood information system. One respondent said, *“Though the medical officers were aware of BIMA but other honorary doctors were not informed about the system*.*”* She also pointed that the information was not conveyed properly to all the doctors who work in obstetric wards.

#### “Necessary Evil”

When participants were asked about the changes of the situation in regards to influence of unlicensed blood brokers after implementation of the BIMA, the respondents indicated that they were still struggling with the problems of unlicensed blood brokers who are barriers to get benefit of BIMA.

In this regard one doctor said, *“It is not possible for us to prevent the broker*. *We need to have strong vigilance team to stop the activity of the broker*. *The doctors cannot stop it*.*”*

Another respondent said, *“BIMA help to save time but number one challenge is the brokers*. *The hospital staff gets percentage from the blood brokers if the patients buy blood from outside*, *they want to make the new system non-functioning*. *It is difficult to sustain the new digital system due to the strong presence of unlicensed blood brokers*.*”*

## Discussion

Although BIMA is an effective approach to get blood during emergency, considerable differences emerged among the different groups of study participants in regards to their perceptions and experiences in the blood transfusion process in a public sector medical college hospital where majority poor patients rush as the last resort for management of PPH. This study explored a strong presence of a cadre of unlicensed blood brokers, who have become de facto, fee-based purveyors of emergent blood. Paying an unlicensed blood broker is often the only way patient attendants can acquire blood, when transfusion has been deemed medically necessary to save life. For example, study participants who are service providers reported that blood is not available in the blood bank and patients do not bring blood donors, so they depend on unauthorized blood centers for blood to save life. The inability of the Bangladeshi health system to fully regulate blood products, and ensure that every CEmOC facility has on-site blood availability has allowed for the development of parallel, un-regulated systems of blood collection.

On the other hand, the attendants of the patient particularly those who are poor and from rural areas don’t know where to go for blood, do not receive any help from hospital so they depend on, and are directed to obtain blood from the unlicensed blood-brokers the quality of which is unknown. Blood-brokers perceive their services as necessary and helpful for patients and income generating for them. Agonizing to note that there is no mechanism to ensure blood product safety in public sector hospitals.

Nobody denies the existence of problems of managing blood, and universal themes included a lack of donors, unsafe blood and the strong presence of blood brokers. Lived experiences of patient’s attendants reveal a wider perspective on the problems than other supply side respondents. The perspective of attendants on the management of blood is based on their experiences as lack of their knowledge of the health system. They identify that they don’t know how to manage blood and there is no support system from the hospital authority. They do not recognize the hazards for unsafe blood transfusion to their patients, when it is collected by the help of the blood broker. When told that they need to obtain blood, they are not given any guidance, nor are there any clear protocols to assist them in their quest. The current system places the responsibility of blood collection upon the shoulders of patient attendants who are unfamiliar with hospital environment, and are not given the tools or information to understand how the needed blood can or should be obtained.

The response from the hospital doctors and managers demonstrates that they have an understanding about the problems but feel they are unable to do anything in the present service delivery contexts. Providers are sympathetic to the patients and they see blood brokers as necessary to save life of a patient. Considering their position in the hospital, they are reluctant to admit their failures and ignorance about the loop holes of the blood management system. The hospital managers and doctors insist that individual behaviors, such as the patients not bringing their own blood donors, as one of the causes of such problems. They overlook factors such as lack of required blood in the blood bank, availability of doctors, their motivations, and lack of information and facilities at the hospital which are key barriers to accessing safe and timely blood. The doctors of blood bank opined that the doctors of the obstetric wards have no coordination with blood bank staffs, and they are uninformed about the availability of required blood in the hospital blood banks.

The most important outcome of the BIMA is it saves time to collect blood and ensures safe blood, but many patients do not know about the new system. The blood bank technologists are not supportive of the digital system as they receive financial benefits from the blood brokers unethically. There is need for technical and motivational training for service providers working both in obstetric wards and blood banks in public hospitals. Strong measures to strengthen health literacy are also needed so that people are willing to donate blood for their near and dear ones. Efforts to ensure safe and timely blood supplies are needed in all facilities in Bangladesh to support achieving Universal Health Coverage (UHC) goals as well as to prevent avoidable maternal deaths.

## Study Limitations

There were few limitations in this study. Getting time from the health service providers to conduct KII was a challenge. We tried to minimize this by doing follow-up visits with the KII participants. This study experienced time constraints due to short study duration as there was formative research prior to the intervention and post intervention qualitative survey. If more time would be provided, it could include observations for triangulation of results obtained through the KIIs and IDIs. A common criticism of qualitative research is ability to generalize the results due to the small sample size. This research does not intend to generalize the result rather tried to gain diverse perspectives from different participants such as health care provider, hospital manager, relatives of the patients, and representative from blood bank and unlicensed blood brokers to understand their views on managing blood for a PPH patient in a particular hospital setting.

## Concluding remarks

Diverse perspectives on blood collection and management are evident from the research. Different views from the different study participants regarding their experience and knowledge on blood collection and management within the Dhaka Medical College hospital provides the necessity to improve the situation to access safe blood for transfusion to ensure no woman dies of a preventable maternal death. The qualitative methods such as key informant and in-depth interviews provided the insight information on respondents’ perspectives, knowledge, experience and it also revealed the attitudes of the different service providers that how they response during the emergency situation to save life of PPH patients when they need blood. The finding of this research shows the strong presence of various realities on blood management in emergency situation. Finally, the findings of this qualitative research provide us a better understanding of the reasons of the facilities’ and families’ dependency on an unregulated and unlicensed cadre of unlicensed blood brokers to access blood to save lives of women. These reasons threatened BIMA as promising intervention to reduce inefficiencies in obtaining blood but steps must be taken to ensure buy-in from current purveyors of blood, and to increase and the acceptance of the intervention. We recommend further interventions to strengthen blood transfusion services in the country.

## Supporting Information

S1 File Appendix A 1st May (PDF)
