# Correction: Barriers to Timely and Safe Blood Transfusion for PPH Patients: Evidence from a Qualitative Study in Dhaka, Bangladesh

**DOI:** 10.1371/journal.pone.0178081

**Published:** 2017-05-16

**Authors:** The PLOS ONE Staff

## Notice of Republication

This article was republished on January 18, 2017, as a Supporting Information file was published in error. The publisher apologizes for the error. Please download this article again to view the correct version.
