# Supplementary material for: Barriers to Timely and Safe Blood Transfusion for PPH Patients: Evidence from a Qualitative Study in Dhaka, Bangladesh
Source: PLoS One. 2016 Dec 2;11(12):e0167399. doi: 10.1371/journal.pone.0167399 (PMC5135104; doi:10.1371/journal.pone.0167399)
Supplement: S1 File — (PDF) [file pone.0167399.s001.pdf]

Appendix A:

A1: Guideline for Key Informants Interview (KII)

1. Background information (name, age, education, religion, designation)

2. What is the present management system of blood transfusion in your hospital?

3. How are you ensuring the supply of sufficient and safe blood for the pregnant and lactating women who come with severe bleeding?

4. What are the challenges/ problems you facing to ensure the supply of blood?

5. How much time is needed to collect blood?

6. How much time is needed to examine the blood group of the patient and donor?

7. What were problems have you had to collect the blood?

8. How did you get the information where and how to get certain group of blood?

If it takes long time what are the options to solve the problems?

9. What is weakness in the current system to examine the blood group and where to get the blood?

10. According to your opinion what type of management/ information can help a patient to get blood quickly at the time of urgent need?

A2: Guideline for In-depth Interview (IDI)

1. Background information (name, age, education, religion, occupation)

2. What was problem with your patient?

3. Where did you take her for treatment?

4. How did you collect blood for her when it was needed?

5.To whom and where did you first go to collect blood?

6. What are the challenges/problems you facing to ensure the supply of blood?

7. How much time was needed to examine the blood group?

8. How much time needed to collect blood?

9. What did the hospital do to get the blood?

10. How much did you spend for it?

11. According to your opinion what type of management/ information can help a patient to get blood quickly at the time of urgent need?
